# Cost-effectiveness of oral ondansetron for children with acute gastroenteritis in primary care: a randomised controlled trial

**DOI:** 10.3399/BJGP.2020.1093

**Published:** 2021-08-24

**Authors:** Anouk AH Weghorst, Gea A Holtman, Irma J Bonvanie, Pien I Wolters, Boudewijn J Kollen, Karin M Vermeulen, Marjolein Y Berger

**Affiliations:** Department of General Practice and Elderly Care Medicine, University of Groningen, University Medical Centre Groningen, The Netherlands.; Department of General Practice and Elderly Care Medicine, University of Groningen, University Medical Centre Groningen, The Netherlands.; Department of General Practice and Elderly Care Medicine, University of Groningen, University Medical Centre Groningen, The Netherlands.; Department of General Practice and Elderly Care Medicine, University of Groningen, University Medical Centre Groningen, The Netherlands.; Department of General Practice and Elderly Care Medicine, University of Groningen, University Medical Centre Groningen, The Netherlands.; Department of Epidemiology, University of Groningen, University Medical Centre Groningen, The Netherlands.; Department of General Practice and Elderly Care Medicine, University of Groningen, University Medical Centre Groningen, The Netherlands.

**Keywords:** acute gastroenteritis, child, cost-effective, ondansetron, primary care, vomiting

## Abstract

**Background:**

Acute gastroenteritis is a common childhood condition with substantial medical and indirect costs, mostly because of referral, hospitalisation, and parental absence from work.

**Aim:**

To determine the cost-effectiveness of adding oral ondansetron to care as usual (CAU) for children with acute gastroenteritis presenting to out-of-hours primary care (OOH-PC).

**Design and setting:**

A pragmatic randomised controlled trial from December 2015 to January 2018, at three OOHPC centres in the north of the Netherlands (Groningen, Zwolle, and Assen) with a follow-up of 7 days.

**Method:**

Children were recruited at the OOH-PC and parents kept a parental diary. Inclusion criteria were: aged 6 months–6 years; diagnosis of acute gastroenteritis; at least four reported episodes of vomiting 24 hours before presentation, at least one of which was in the 4 hours before presentation; and written informed consent from both parents. Children were randomly allocated at a 1:1 ratio to either CAU (oral rehydration therapy) or CAU plus one dose of 0.1 mg/kg oral ondansetron.

**Results:**

In total, 194 children were included for randomisation. One dose of oral ondansetron decreased the proportion of children who continued vomiting within the first 4 hours from 42.9% to 19.5%, (a decrease of 54.5%), with an odds ratio of 0.4 (95% confidence interval [CI] = 0.2 to 0.7; number needed to treat: four). Total mean costs in the ondansetron group were 31.2% lower (€488 [£420] versus €709 [£610]), and the total incremental mean costs for an additional child free of vomiting in the first 4 hours was −€9 (£8) (95% CI = −€41 [£35] to €3 [£3]).

**Conclusion:**

A single oral dose of ondansetron for children with acute gastroenteritis, given in OOH-PC settings, is both clinically beneficial and cost-effective.

## INTRODUCTION

The high incidence of acute gastroenteritis among children aged <5 years in the Netherlands (609 per 1000 person–years) is associated with substantial medical and indirect costs.^1^^,^^2^ The total costs in this age group are estimated at €77.28 million (£66.5 million) per year.^3^ Referral to specialist care — and hospitalisation in particular — are the main drivers of high medical costs,^4^ but hospitalisation results in parents missing work, which also contributes to high indirect costs.^5^

Acute gastroenteritis usually has a self-limiting course in children.^1^ Oral rehydration therapy (ORT) is recommended for mild-to-moderate dehydration, but it remains underused.^2^^,^^6^ Excessive vomiting during acute gastroenteritis can cause ORT failure, which in turn, can be responsible for referral and hospitalisation.^7^ Symptomatic treatment of vomiting may, therefore, prevent ORT failure, reduce referral rates to emergency departments, and decrease medical and indirect costs.^8^^–^^11^ The most widely used antiemetics to date — domperidon and metoclopramide — are not recommended overall because of a lack of evidence of their effectiveness and the risk of severe side-effects;^6^^,^^12^ the *Dutch Paediatric Formulary* recommends oral ondansetron for children with acute gastroenteritis, vomiting, and dehydration.^13^ Ondansetron, a 5-HT_3_ serotonin antagonist with a central antiemetic effect, has not only been shown to decrease vomiting rates by 54.5% among children at increased risk of dehydration in out-of-hours primary care (OOH-PC) settings, it also seems to be safe and positively evaluated by parents.^14^ Its use reduces immediate hospitalisation rates and the need for intravenous rehydration therapy, while enhancing compliance with ORT;^7^^,^^15^ in addition, no serious adverse events have been reported to date.^15^^,^^16^

Despite the available data in support of the clinical efficacy of ondansetron, data are lacking about the cost-effectiveness of adding ondansetron to care as usual (CAU) in OOH-PC settings. Cost-effective data are used, in addition to clinical evidence, in decision making by policymakers and guideline developers. Therefore, the aim was to assess the cost-effectiveness of adding oral ondansetron to CAU in children aged 6 months–6 years with acute gastroenteritis in OOH-PC settings.

## METHOD

### Design and setting

The cost-effectiveness of adding oral ondansetron to CAU was studied alongside a randomised controlled trial (RCT) on the effectiveness of this approach. The RCT started with a pilot study (NL4700) (https://www.trialregister.nl/trial/4700) that was carried out from December 2015 until October 2016, and then extended with the final trial until January 2018; it was conducted at three OOH-PC centres in the north of the Netherlands (Groningen, Zwolle, and Assen). The design, recruitment strategy, outcomes, and informed-consent procedure of the RCT are reported elsewhere.^17^ In agreement with the Medical Ethics Review Committee of the University Medical Center Groningen, the primary outcome changed from referral to vomiting to guarantee an outcome that was more relevant to patients. The researchers were allowed to include children from the pilot study in the final trial (NL5830) (https://www.trialregister.nl/trial/5830).

**Table:** How this fits in

Ondansetron has already been shown to effectively reduce vomiting in children with acute gastroenteritis who are at increased risk of dehydration. This study reveals that a single dose of oral ondansetron to care-as-usual at the out-of-hours primary care service also decreases the total mean costs of managing acute gastroenteritis in these children by 31.2% from €709 (£610) to €488 (£420). Implementation of oral ondansetron in primary care would, therefore, not only be clinically beneficial but also cost-effective.

### Participants

Children aged 6 months–6 years with a diagnosis of acute gastroenteritis who were considered to be at increased risk of dehydration were included,^12^ based on the following inclusion criteria:

- at least four episodes of vomiting 24 hours before presenting to the OOH-PC centre;
- at least one episode of vomiting in the 4 hours before presenting to the OOH-PC centre; and
- written informed consent of both parents.

The age range of 6 months–6 years was chosen for two reasons: the known incidence of acute gastroenteritis and related dehydration is highest in children aged <6 years old;^9^ and, as an age of <6 months is seen as an additional risk factor for ORT failure at home, Dutch paediatric and GP guidelines recommend low-threshold referral in children aged <6 months and at risk of dehydration.^12^^,^^18^

The exclusion criteria were as follows:

- antiemetic use or prescription in the 6 hours before presentation;
- known renal failure or hypoalbuminemia;
- known diabetes mellitus or inflammatory bowel disease;
- history of abdominal surgery explaining current symptoms according to the GP;
- known sensitivity to 5-HT_3_ receptor antagonists;
- known prolonged QT interval or current use of QT-prolonging medication; and
- previous enrolment in the study.

### Randomisation and blinding

Children were randomly allocated to one of two intervention groups at a 1:1 ratio. An online randomisation tool generated the allocation sequence in direct response to participant inclusion by the research assistant. Allocation was not generated before inclusion to ensure concealment, and the allocation sequence was stratified by age (6–24 months or >24 months) and dehydration severity (‘at risk’, meaning no alarm symptoms; or ‘dehydrated’, meaning at least one alarm symptom). Risk factors assessed at baseline were: ≥6 watery stools or diarrhoea, fever, and reduced intake. The following alarm symptoms were assessed at baseline:

- confused or decreased consciousness;
- bradycardia;
- weak peripheral pulses;
- capillary-refill time of >4 seconds;
- skin-pinch test of >4 seconds;
- cold or marbled extremities; and
- no urine output for 24 hours.

This study was designed as a pragmatic RCT with emphasis on the potential implementation of ondansetron in primary care, so participants, parents, GPs, and research assistants were deliberately not blinded to treatment allocation. In this case, blinding participants would result in outcomes that could not be translated to daily practice. The statistician, who performed the statistical analyses was blinded to treatment allocation; an independent statistician performed this blinding. The primary outcome was not known by participants, parents, or GPs.

### Interventions

#### Control group, CAU

CAU involved giving instruction on the use of ORT, as described in the guideline for acute diarrhoea by the Dutch College of GPs.^12^ This included advice to buy an oral rehydration solution, together with the following instructions on how to use it: 10 mL/kg compensation for diarrhoea when at risk (that is, all children) and 15 mL/kg for 4 hours if assessed as dehydrated by the GP. The research assistant provided the instructions, together with a patient folder in which the information was repeated. In addition, the research assistant discussed alarm symptoms and advised parents to contact the GP if there was either no improvement or a worsening of symptoms 4 hours after presentation.

ORT had to be bought by parents at the pharmacy or over the counter, and was initiated at home. If children were referred to the hospital within 1 hour after randomisation, the CAU was considered as not received and were removed from the per protocol analysis in the effectiveness outcome.

#### Intervention: CAU plus ondansetron

Children allocated to the intervention group received a single weight-based dose of oral ondansetron syrup (0.1 mg/kg body weight) in accordance with the *Dutch Paediatric Formulary* .^13^ If the child vomited within 15 minutes after administration, this dose was repeated once.

Ondansetron therapy was considered ‘received’ if one adequate dose had been successfully administered within 1 hour after randomisation. So if children were referred within 1 hour, it was noted as ‘not received‘.

### Follow-up

Parents were asked to complete a diary for 7 days. In the first 4 hours, they were asked to report on their child’s progress each hour; thereafter, they reported once daily until 7 days after presentation.

The primary outcome was assessed on return of the diary or by telephone if parents had not returned the diary after three requests.

### Outcomes

#### Primary outcome

The efficacy of the study medication, assessed as the proportion of children who continued vomiting in the first 4 hours after randomisation (that is, at least one episode), has been reported previously.^14^ The fourth hour was considered based on two criteria: national guidelines, which state that GPs should re-evaluate dehydrated children after 4 hours;^11^ and the circulating concentration of ondansetron, which is expected to reach 50% of its maximum serum level at 3 hours after oral ingestion^19^ (the half life of ondansetron is 3 hours, which is used to examine the effect).

#### Costs

Costs were grouped into healthcare and indirect costs (see Supplementary Table S1). They were valued according to the cost manual of the National Health Care Institute of the Netherlands^20^ and the standard prices of the medication.^21^ Prices were indexed to the level of 2018 and are reported in euros. The measurements for the cost analyses were based on the details provided in the parental diaries.

### Statistical analysis

The total mean cost and effectiveness per group were compared based on complete cases. To be eligible for analysis, each child needed complete data on cost and effect. Comparing the demographic characteristics of children with and without complete cost-and-effect pairs suggested data were missing at random. A cost-effectiveness analysis was then performed, in which the effect of ondansetron added to CAU was compared with CAU alone. The primary outcome measure (unit of health) was the number of children who continued to vomit within 4 hours; the time horizon for the analysis was 7 days.

Incremental costs and outcomes were assessed, and are expressed as an incremental cost-effectiveness ratio, representing the additional costs or savings per additional child free of vomiting. Any difference in effect, based on the primary outcome, was divided by the cost difference between interventions. Cost-and-effect pairs were bootstrapped (5000 replications) to calculate alternate confidence intervals (CIs) and plotted on a cost-effectiveness plane. In addition, a cost-effectiveness acceptability curve (CEAC) was plotted to evaluate the probability that adding a single dose of oral ondansetron to CAU is more cost-effective than CAU alone, over a range of different maximum values. This was used to reveal whether the intervention was cost-effective compared with CAU over a range of maximum monetary values that a decision maker may be willing to pay for an additional unit of health.^22^

## RESULTS

### Study sample

**Figure 1.**
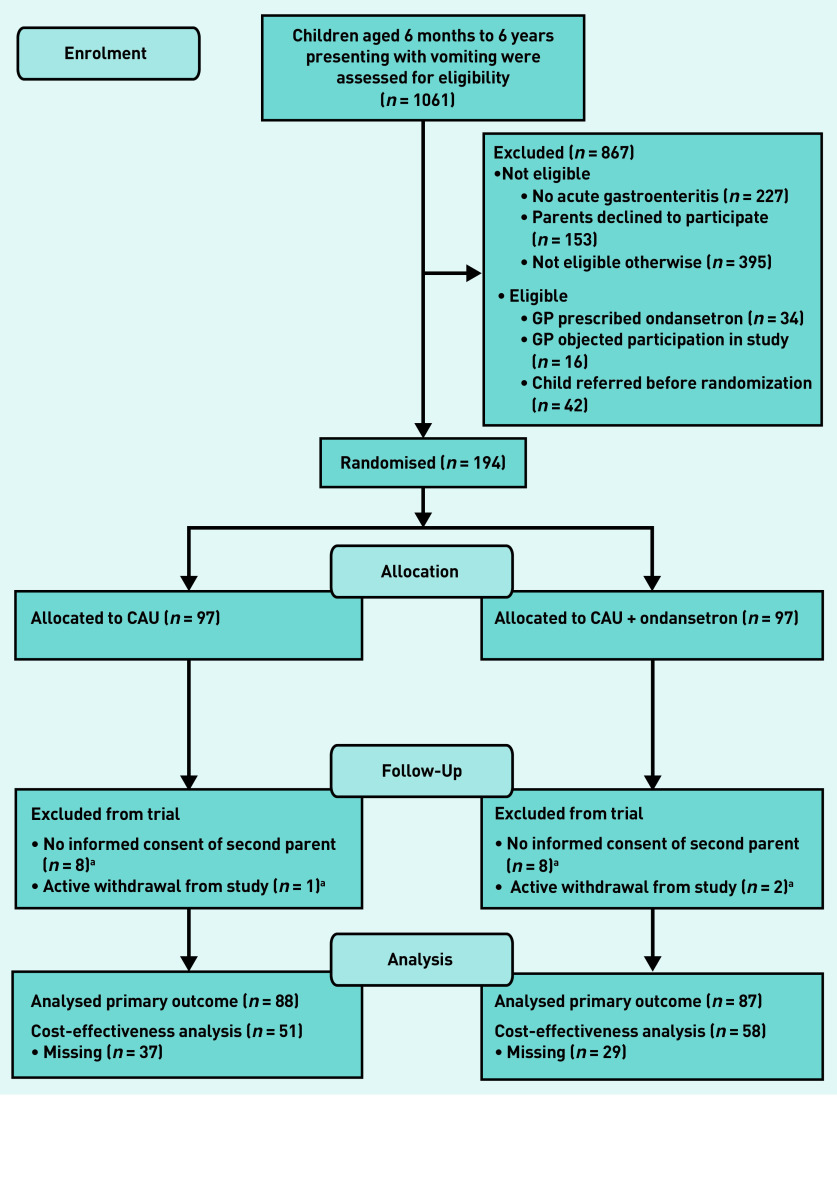
***Study flow diagram.*** *^a^* ***Excluded from trial because of no informed consent of second parent or active withdrawal from study (retracted informed consent). CAU = care as usual.***

The study process is summarised in Figure 1. A total of 1061 children were screened for eligibility at the participating OOH-PC centres. Of the 867 children who were excluded, 775 were ineligible. This was because they were assessed as not being at increased risk of dehydration (*n* = 395), did not have a diagnosis of acute gastroenteritis (*n* = 227), and the parents declined to participate (*n* = 153).

In total, 194 children were included, with 97 each allocated randomly to the control and intervention groups (Figure 1). Another 19 children were excluded after randomisation because no second written informed consent was obtained (*n* = 16) or they withdrew from the study (*n* = 3), (data not shown).

Data for 175 children (*n* = 88 CAU, *n* = 87 intervention) were then available for analysis of the primary efficacy outcome (Figure 1). Data for 109 children were available for the cost-effectiveness analysis (*n* = 51 control, *n* = 58 intervention).

### Baseline characteristics of included participants

Of the included participants, the median age was 1.5 years (range: 6 months–6 years, medium IQR), 50.3% were female, the median duration of vomiting before presentation was 2 days (range: 0.8–9.0 days, medium IQR), and 71.3% experienced diarrhoea (*n* = 124).

There were no major differences in baseline characteristics between children in the control and intervention groups (Table 1).

**Table 1.** Baseline characteristics of the population for all participants (*N* = 175), the control group (*n* = 88), and the intervention group (*n* = 87)

**Characteristics**	**Valid responders, *n***	**All participants**	**Valid responders, *n***	**Control group**	**Valid responders, *n***	**Intervention group**
**Age, years, median (IQR)**	175	1.5 (0.9–2.1)	88	1.5 (0.9–2.0)	87	1.5 (0.9–2.2)

**Female, *n* (%)**	175	88 (50.3)	88	50 (56.8)	87	38 (43.7)

**Weight, kg, median (IQR)**	169	11.0 (9.5–14.0)	86	11.0 (9.4–14.0)	83	12.0 (9.5–14.3)

**Duration of vomiting prior to presentation, days, median (IQR)**	174	2.0 (1.0–3.0)	87	1.2 (1.0–2.0)	87	2.0 (1.0–3.0)

**Frequency of vomiting in past 24 hours, median (IQR)**	171	5.0 (4.0–10.0)	86	5.0 (4.0–10.0)	85	6.0 (4.0–10.0)

**Diarrhoea present, *n* (%)**	174	124 (71.3)	87	66 (75.9)	87	58 (66.7)

**Duration of diarrhoea prior to presentation, days, median (IQR)^a^**	124	2.0 (1.0–3.0)	66	1.0 (0.4–2.0)	58	1.0 (0.0–3.0)

**Frequency of diarrhoea in past 24 hours, median (IQR)^a^**	123	3.0 (2.0–5.0)	66	2.0 (1.0–5.0)	57	1.5 (0.0–4.0)

**Dehydration assessed at 0–100% by GP, median (IQR)**	170	20.0 (10.0–40.0)	85	20.0 (6.0–40.0)	85	20.0 (10.0–40.0)

**Use of concomitant medication, *n* (%)**	175	65 (37.1)	88	31 (35.2)	87	34 (39.1)

**Additional risk factors of dehydration, *n* (%)^b^**						
1	175	63 (36.0)	88	33 (37.5)	87	30 (34.5)
≥2	175	18 (10.3)	88	10 (11.4)	87	8 (9.2)

**Alarm symptoms of severe dehydration, *n* (%)^c^**						
1	175	32 (18.3)	88	15 (17.0)	87	17 (19.5)
≥2	175	2 (1.1)	88	1 (1.1)	87	1 (1.1)

a *Numbers only presented for those participants with diarrhoea.*

b *Risk factors assessed at baseline were:* ≥*6 watery stools or diarrhoea, fever, and reduced intake of liquid/food.*

c *Alarm symptoms assessed at baseline were: confused or decreased consciousness, bradycardia, weak peripheral heartbeat pulsations, capillary refill time* >*4 seconds, skin pinch test* >*4 seconds, cold or marbled extremities, and no urine output in the previous 24 hours. IQR = interquartile range.*

### Health outcomes

One dose of oral ondansetron decreased the proportion of children who continued vomiting within the first 4 hours from 42.9% (*n* = 33/77) to 19.5% (*n* = 15/77). The odds ratio for this association was 0.4 (95% CI = 0.2 to 0.7), giving a number needed to treat of four.^14^

### Cost-effectiveness analysis

Costs for the control and intervention groups are outlined in Table 2. The total mean costs in the intervention group (€488 [£420]) were 31.2% lower (mean difference €221 [£190]) than in the CAU group (€709 [£610]). Total healthcare costs per patient were also lower in the intervention group, by €48 (£41), with hospital admission being the main driver. The costs for hospital admission were also calculated per day, meaning that children in the CAU group were admitted to hospital for longer. Indirect costs (that is, work absence of parents) accounted for 62.9% (€446 [£384]) of the total costs in the CAU group and 55.7% (€272 [£234]) in the intervention group, giving a reduction of €174 (£150).

**Table 2.** Total mean costs for the control (*n* = 51) and intervention groups (*n* = 58)

**Cost type**	**Control**	**Intervention**
**Healthcare costs in** €**, mean (SD)**		
General practice	54 (93)	40 (64)
OOH-PC	1 (5)	2 (8)
Referral to pediatrician	45 (72)	37 (74)
Hospital admission	162 (512)	134 (426)
Oral rehydration solution	2 (3)	3 (3)

**Indirect costs in** €**, mean (SD)**		
Work absence, mother	287 (390)	151 (216)
Work absence, father	159 (258)	121 (274)

**Total costs all sectors in** €**, mean (SD)**	709 (839)	488 (638)

*OOH-PC = out-of-hours primary care. SD = standard deviation.*

**Figure 2.**
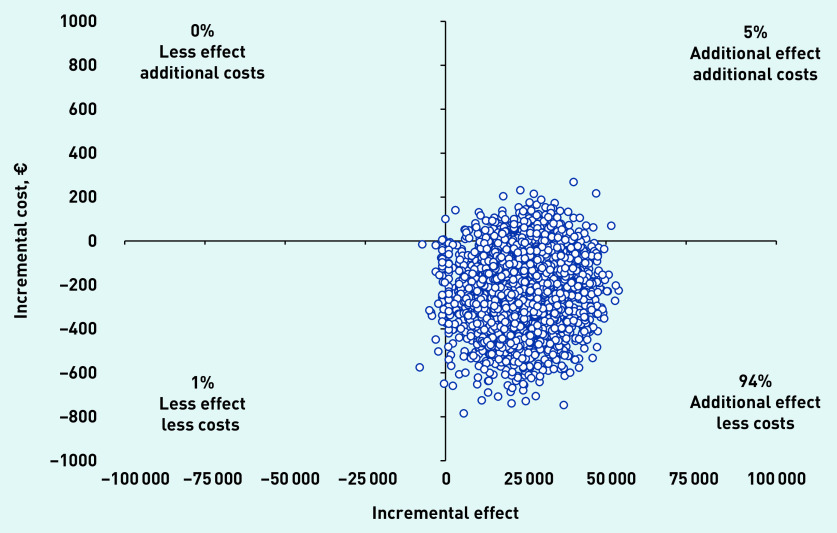
*Cost-effectiveness plane.*

**Figure 3.**
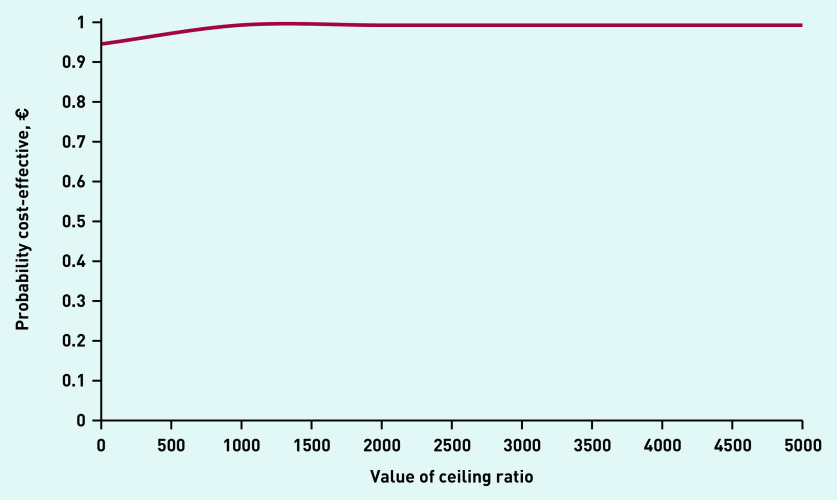
*Cost-effectiveness acceptability curve.*

The total incremental mean cost per child free of vomiting within 4 hours of assessment was −€9 (£8) (95% CI = −€41 to €3) The cost-effectiveness plane revealed 94.0% of the bootstrap replicates to be in the bottom-right quadrant, indicating lower costs and better effectiveness with ondansetron (Figure 2). The CEAC indicated an almost 95% chance that the intervention was cost-effective without investing additional money; however, at an investment of approximately €1000, the chance of the intervention being cost-effective increased to 100% (Figure 3).

## DISCUSSION

### Summary

This RCT showed the cost-effectiveness of adding a single dose of oral ondansetron to CAU for children at increased risk of dehydration due to acute gastroenteritis in an OOH-PC setting. Specifically, one dose of ondansetron was associated with a decrease in the percentage of children with persistent vomiting due to acute gastroenteritis over the first 4 hours after assessment from 42.9% to 19.5%, saving an average of €9 (£8) per child who stopped vomiting. The total mean costs were 31.2% lower with the addition of ondansetron, making it a cost-effective treatment for children diagnosed with acute gastroenteritis in OOH-PC settings.

### Strengths and limitations

This is the first study, to the authors’ knowledge, to evaluate the cost-effectiveness of adding oral ondansetron to CAU when managing acute gastroenteritis among children in OOH-PC centres. Nearly 600 GPs collaborated and nearly all children aged6 months–6 years who presented with vomiting at three OOH-PCs in the north of the Netherlands over a period exceeding 2 years were screened. As such, the sample is highly representative of children presenting to OOH-PC centres at increased risk of dehydration. Patients seen in the three centres were representative of the general population. Moreover, the use of an hourly diary for the first 4 hours, and a daily dairy for another 7 days, provided important follow-up data. Another strength is that the findings were based on estimated healthcare utilisation and associated costs from the National Health Care Institute of the Netherlands^20^ and the standard prices of the medication costs,^21^ indexed to 2018; these ensure the data are representative and applicable for decision makers overall.

This study also has some limitations. Data were available for 109 participants (62.3% of included children) only, when calculating the total mean costs; however, bootstrapping (5000 replications) meant that accounting for the missing data did not alter the findings. Participants, parents, GPs, and research assistants were not blinded to the intervention but, given the pragmatic design, it is contentious whether this would have been desirable. Ondansetron has already been proven effective at reducing vomiting in blinded RCTs in specialist care^23^^,^^24^ and, aside from the research assistants, the groups were unaware of the primary outcome. Parents were informed about ondansetron and that the course of acute gastroenteritis was being investigated but, as no information was given regarding a specific focus on vomiting, the authors do not think the lack of blinding affected the study’s outcomes.

Another limitation is that only work absence by parents was considered in the indirect costs, with other non-medical costs — such as consumption of special food, extra diaper use, and travel costs — excluded. This choice was deliberate to avoid burdening the parents of sick children with excessive information requests; however, absence from work is known to be the largest contributor to indirect costs when managing children with acute gastroenteritis.^3^^,^^5^ The costs of oral ondansetron were also not included; this was because these are extremely low (€0.25–€0.37 [per dose]).

### Comparison with existing literature

The study presented here showed that an average of €9 (£8) could be saved for every additional child who did not vomit in the first 4 hours after being given a single dose of ondansetron. With an incidence of 1.96 episodes/person–years and an average annual cost of €88.57 (£76) per child aged <5 years, oral ondansetron could lead to significant cost reductions.^3^

The main cost drivers in the study presented here — hospitalisation and work absence — were comparable with those reported in another study.^3^ The differences in costs between groups can be explained by the reductions in health care and indirect costs with ondansetron use, resulting in fewer referrals to a paediatrician and fewer hospital admissions, which typically drive costs, as stated by Elliott.^25^

Paediatrician referrals were made for 19% of children in the present study, far higher than the previously reported rate of 8%,^26^ but these almost certainly resulted from the deliberate inclusion of children at increased risk of dehydration; supporting this, the degree of dehydration is known to be among the main reasons for referral and hospitalisation.^27^

The costs for hospital admission were also calculated per day, so the results showed that children in the control group were admitted to hospital for longer. Furthermore, costs for a GP visit were lower in the intervention group, indicating that these children were less likely to require a repeat visit to the GP. These results imply that adding oral ondansetron to CAU could reduce the considerable burden that acute gastroenteritis places on the healthcare system in the Netherlands.^2^

Differences in indirect costs were attributable to fewer work absences in the intervention group. This was particularly evident for mothers of children not receiving ondansetron, among whom productivity losses are typically double those of fathers, and consistent with evidence that mothers stay at home more often than fathers to take care of sick children.^28^ In the US, 80% of non-medical costs per case of acute gastroenteritis in children were shown to be attributable to parents missing work.^29^ In the CAU group in the study presented here, parental work absence accounted for 62.9% of the total costs compared to 55.7% in the ondansetron group. Work absence also tends to increase with the severity of acute gastroenteritis (that is, degree of dehydration);^30^ the parents of children who received ondansetron required less time off work because of their sick child and, as a consequence, had lower indirect costs.

### Implications for practice

A single dose of oral ondansetron is cost-effective for children who are at increased risk of dehydration and present to OOH-PC with vomiting due to acute gastroenteritis. Multiple studies have proven the efficacy and safety of oral ondansetron in emergency departments. The authors recommend advocating oral ondansetron use in primary care guidance on the management of vomiting in children with acute gastroenteritis who are at increased risk of dehydration; this could reduce both the burden of the disease for children and the costs to the healthcare system and wider society.
